# Human adipose derived mesenchymal stromal cells transduced with GFP lentiviral vectors: assessment of immunophenotype and differentiation capacity in vitro

**DOI:** 10.1007/s10616-016-9945-6

**Published:** 2016-01-27

**Authors:** Fiona A. van Vollenstee, Carlo Jackson, Danie Hoffmann, Marnie Potgieter, Chrisna Durandt, Michael S. Pepper

**Affiliations:** 1 Department of Immunology, Faculty of Health Sciences, Institute for Cellular and Molecular Medicine and MRC Extramural Unit for Stem Cell Research and Therapy, University of Pretoria, P.O. Box 2034, Pretoria, 0001 South Africa; 2 Plastic and Reconstructive Surgeon, Private Practice, Pretoria, South Africa

**Keywords:** Adipose derived stromal/stem cells (ASCs), ASC labeling, Green fluorescent protein (GFP), Lentiviral vector, Viral transduction

## Abstract

Adipose derived mesenchymal stromal/stem cells (ASCs) are a heterogeneous population characterized by (a) their ability to adhere to plastic; (b) immunophenotypic expression of certain cell surface markers, while lacking others; and (c) the capacity to differentiate into lineages of mesodermal origin including osteocytes, chondrocytes and adipocytes. The long-term goal is to utilize these cells for clinical translation into cell-based therapies. However, preclinical safety and efficacy need to be demonstrated in animal models. ASCs can also be utilized as biological vehicles for vector-based gene delivery systems, since they are believed to home to sites of inflammation and infection in vivo. These factors motivated the development of a labelling system for ASCs using lentiviral vector-based green fluorescent protein (GFP) transduction. Human ASCs were transduced with GFP-expressing lentiviral vectors. A titration study determined the viral titer required to transduce the maximum number of ASCs. The effect of the transduced GFP lentiviral vector on ASC immunophenotypic expression of surface markers as well as their ability to differentiate into osteocytes and adipocytes were assessed in vitro. A transduction efficiency in ASC cultures of approximately 80 % was observed with an MOI of ~118. No significant immunophenotypic differences were observed between transduced and non-transduced cells and both cell types successfully differentiated into adipocytes and osteocytes in vitro. We obtained >80 % transduction of ASCs using GFP lentiviral vectors. Transduced ASCs maintained plastic adherence, demonstrated ASC immunophenotype and the ability to differentiate into cells of the mesodermal lineage. This GFP-ASC transduction technique offers a potential tracking system for future pre-clinical studies.

**Electronic supplementary material:**

The online version of this article (doi:10.1007/s10616-016-9945-6) contains supplementary material, which is available to authorized users.

## Introduction

Adipose derived mesenchymal stromal/stem cells (ASCs) are a heterogeneous cell population characterized (a) by their ability to adhere to plastic; (b) the immunophenotypic expression of certain cell surface markers (CD73, CD90, CD105) while lacking others (CD34, CD45); and (c) the capacity to differentiate into cells of mesodermal origin including osteocytes, chondrocytes and adipocytes (Bunnell et al. 2008; Dominici et al. 2006; Pittenger et al. 1999). This makes ASCs an attractive option for tissue engineering and regenerative medicine. Pre-clinical studies using experimental animal models to establish safety and efficacy are an essential requirement before ASCs can confidently be assessed as cellular therapy products in clinical trials. However, the unavailability of efficient and reliable methods of tracking and monitoring ASCs in vivo constitutes a significant obstacle.

Green fluorescent protein (GFP) has previously been used to evaluate living cells in situ (Kafri et al. 1997; Miyoshi et al. 1997; Naldini et al. 1996; Tao et al. 2014) as it emits fluorescence that can be detected by most microscopy as well as flow cytometry systems. For this reason it is also an attractive marker for in vivo tracking of stem cells in animal models. It is however immunogenic which may compromise the interpretation of results (Yang et al. 2014).

Lentiviral vectors are gene delivery tools which have the ability to integrate genetic cargo into the chromosomes of a target cell. This allows for the delivery of most forms of genetic material while minimizing the risk of the vector-transduced cells being attacked by virus-specific cytotoxic T-lymphocytes. This is accomplished by not transferring sequences encoding for proteins required for packaging the virus (Naldini et al. 1996; Barde et al. 2010).

ASCs can be utilized as biological vehicles for vector-based gene delivery systems, since they are believed to home to sites of inflammation and infection in vivo (Karp et al. 2009; Sordi et al. 2005). The approach to lentiviral transduction optimization for gene therapy applications must be distinguished from transduction efficacy used to produce an efficient tracking system. Integration of the lentiviral genomic material into the target cell genome is dependent on lentiviral integrase, the key determinant for gene-targeted integration specificity (Lewinski et al. 2006). Human immunodeficiency virus type-1 (HIV-1)-based lentiviral vectors mediate efficient gene transfer to both proliferating and quiescent cells, although transgene insertion into the DNA of target cells is biased toward transcriptionally active loci, increasing the risk of insertional mutagenesis (Staunstrup et al. 2009). Although the integration of one lentiviral genome copy per target cell would be ideal for the application of gene therapy, thereby decreasing the risk of mutagenesis, multiple copies per target cell would be preferable for tracking purposes. The presence of multiple copies per cell results in strong GFP expression and consequently a high fluorescence signal which would make tracking easier and more efficient. However, care should be taken that insertion of multiple GFP copies per cell does not change the basic characteristics of the cell and result in cell transformation.

The aim of this study was to optimize GFP transduction efficiency of human ASCs using lentiviral vectors and to evaluate the effect of transduction on ASC immunophenotype and differentiation into various lineages.

## Materials and methods

### Study setting

Approval was obtained from the Research Ethics Committee of the Faculty of Health Sciences, University of Pretoria (protocol number 218/2010). Written informed consent was obtained prior to lipoaspirate harvesting from healthy donors undergoing routine plastic or reconstructive surgery procedures.

### Isolation, characterization and expansion of ASCs

Human ASCs were isolated from lipoaspirate, and were characterized and expanded according to protocols described by Zuk (2001) and Bunnell et al. (2008; Zuk 2001). In short, lipoaspirate was washed three times with phosphate-buffered saline (PBS) to remove contaminating peripheral blood cells, after which the sample was subjected to enzymatic digestion to separate the stromal vascular fraction (SVF) from mature adipocytes. The red blood cells were lysed and the cell suspension strained through a 70 µm cell strainer (Millipore, Billerica, MA, USA) before seeding the SVF into culture flasks at a density of 5.0 × 10^5^ cells/cm^2^. After 24 h the non-adherent cells were removed resulting in selection for the adherent cell component present in the adipose derived SVF. ASC cultures were maintained under standard culture conditions (37 °C, 5 % CO_2_) in alpha-Modified Eagle Medium GlutaMax™ culture medium (α-MEM, Gibco, Life Technologies/Thermo Fischer Scientific, Carlsbad, CA, USA) supplemented with 10 % fetal bovine serum (FBS) and 1 % penicillin and streptomycin (pen/strep, Gibco). At 80 % confluence, the cultures were passaged using 0.25 % Trypsin/EDTA (Gibco) and re-seeded at a density of 5.0 × 10^3^ cells/cm^2^.

### Preparation of GFP encoding lentiviral vectors

The three plasmids used for lentiviral vector production were psPAX2 (envelope function), pMD2G (packaging function) and pLVTH (transfer function). The pLVTH plasmid is a self-inactivating vector (SIN) using an EF1α promotor to drive GFP expression (Wiznerowicz and Trono 2003).

Vector stock was obtained through heat shock transformation of DH5α bacteria and subsequent extraction from the cells using a Zyppy plasmid Maxiprep Kit (Zymo Research Corporation, Irvine, CA, USA). The extracted plasmids were concentrated to 1 µg/µl and stored at −20 °C.

Plasmids were co-transfected into the human embryonic kidney 293T cell line (HEK 293T cells; NIH AIDS Reagent Program, Germantown, MD, USA) to produce lentiviruses that are capable of transducing the GFP gene as previously described by Barde et al. (2010).

Three culture dishes (100 mm diameter) were seeded with HEK 293T cells at 1–3 million cells per dish in 10 ml Dulbecco’s Modified Eagle Medium (DMEM) supplemented with 10 % FBS and 1 % pen/strep. Plasmid DNA concentrations were 1 µg/µl, and the amounts used for transfecting 293T cells were 3 µl pMD2G, 8 µl psPAX2 and 10 µl pLVTH, respectively. The plasmids were resuspended in double distilled water (ddH_2_O) containing 2.5 mM Hepes and 250 μl of a 0.5 M CaCl_2_ solution. One millilitre of the mixture was added to the pre-prepared HEK 293T cell culture dishes followed by incubation for 12 h under standard culture conditions. After 12 h the conditioned medium was aspirated, followed by a washing step with PBS supplemented with 1 % pen/strep after which 15 ml DMEM was added to the culture. The cultures were incubated for an additional 24 h under standard culture conditions.

The conditioned medium (supernatant; 15 ml) containing the lentiviral stock was harvested from the cultures. The first harvest pool was placed into 50 ml tubes and stored at 4 °C. The harvesting procedure was repeated twice. The pooled supernatant was then centrifuged for 10 min at 1153*g* to produce a pellet consisting of cells and debris. The cell-free supernatant was subjected to ultra-centrifugation for 120 min (16 °C) at 49,460*g* after which the supernatant was discarded and the pellet re-suspended in 80 μl Hank’s Balanced Salt Solution without Ca_2_^+^Mg_2_^+^ (HBS). The vector stock/HBS solution was vortexed gently every 30 min while being incubated at room temperature for 2 h. The vector stock solutions from three transfection procedures were pooled to produce a homogenous vector stock solution and stored at −80 °C. Freezing and thawing of the lentiviral stock were avoided as far as possible.

### Optimization of ASC transduction by the GFP encoding lentiviral vectors

For tracking purposes, a titration study was performed to establish a linear regression standard curve as well as the optimal titer that would lead to the maximum number of cells in the ASC population expressing GFP (Supplementary Figure 1). ASCs were seeded into two 6-well plates at a density of 5 × 10^3^ cells per cm^2^ and incubated for 12 h under standard culture conditions. Varying amounts of vector stock were added to the wells containing adherent ASCs in 2 ml DMEM supplemented with 1 % pen/strep and 10 % FBS (Table 1). The control (non-transduced) wells received 250 μl sterile PBS. The plates were maintained under standard culture conditions, and the DMEM plus 1 % pen/strep and 10 % FBS was replaced every 48 h until 80–90 % confluence was reached. The cultures were passaged by trypsinization using 0.25 % Trypsin/EDTA (Gibco). Enzymatic activity was halted after 20 min through the addition of 2 ml DMEM plus 1 % pen/strep and 10 % FBS and the cell suspension centrifuged at 265*g* for 5 min at 21 °C. The pellet was re-suspended in 1 ml PBS plus 2 % pen/strep and a 100 μl aliquot was used for GFP expression analysis using flow cytometry (Gallios, Beckman Coulter, Miami, FL, USA). The remaining cell suspension was re-seeded into culture at a density of 5 × 10^3^ cells/cm^2^. The number of cells expressing GFP was measured for all the respective titration cultures over 10 passages. The D’Agostino & Pearson omnibus normality test was used to assess for a Gaussian distribution over the 10 passages at the various exposure concentrations.

**Table 1:** Volume of vector stock added to 48,000 ASCs seeded 12 h prior to transduction, with the mean percentage cells expressing GFP across 10 post-transduction passages and respective MOIs

Amount vector stock (μl) per well	Mean % ASCs expressing GFP^a^	MOI
0 μl (150 μl PBS)	0.90	0
0 μl	0.61	0
5 μl	2.71	2
25 μl	8.41	12
50 μl	5.25	24
100 μl	11.32	47
150 μl	56.93	71
200 μl	56.20	94
250 μl	74.85	118
300 μl	75.73	141

^a^ASCs were from biological replicate no 1. These data were however only for titration purposes only

### ASC transduction with GFP encoding lentiviral vectors

ASC cultures from three different individual donors were characterized according to the criteria set out by Dominici et al. (2006) before they were considered for the transduction experiments. The three cultures at passages 8, 11 and 14 were each seeded separately into 2 wells of a 6-well plate containing 2 ml DMEM plus 1 % pen/strep and 10 % FBS at a seeding density of 5 × 10^3^ cells/cm^2^, and incubated for 12 h under standard culture conditions. The titration experiment demonstrated transduction of >74 % of the ASC population based on mean GFP expression. This was maintained over 10 consecutive passages post transduction. 250 µl of viral stock solution resulted in a titer of 22,594 transducing units (TU)/μl and an MOI ~118. The control wells (non-transduced) from the same individual ASC culture received 250 μl of PBS. The cultures were maintained under standard culture conditions and DMEM plus 1 % pen/strep and 10 % FBS was replaced every 48 h. The non-transduced and the transduced cells were treated in exactly the same way for all purposes and for each individual ASC culture, and were maintained under similar conditions and passaged at the same time.

### Evaluation of transduction efficacy and immunophenotype using flow cytometry

The transduced and non-transduced cells from an individual ASC culture were trypsinized and re-suspended in PBS. A 100 μl aliquot from both cell suspensions was simultaneously stained with a panel of monoclonal mouse anti-human antibodies (CD34, CD45, CD73, CD90 and CD105; Beckman Coulter, Miami, FL, USA). Another 100 μl from the non-transduced cell suspension was used as the unstained immunophenotypic control (GFP negative control). The cell suspensions were incubated in the dark at room temperature for 10 min before washing three times with PBS supplemented with 10 % FBS and 1 % pen/strep. The GFP positive population was detected in the FL1 channel (emission spectrum; 525/40 nm) and the GFP positive ASC population was calculated as a percentage of viable ASCs. Cell populations were immunophenotypically characterized by determining the expression or lack of expression of respective cell surface markers on a single cell basis.

### Lineage induction and qualitative capacity assessment of transduced and non-transduced ASCs

Both the transduced and non-transduced cultures were induced to differentiate into adipogenic and osteogenic lineages. The adipogenic and osteogenic induction protocols were adapted from methods described previously by Zuk et al. (2002) and Zuk (2001). Oil Red O was used to qualitatively assess the adipocyte differentiation capacity by visualizing lipid droplets in mature adipocytes. Osteogenic differentiation was qualitatively assessed using 2 % Alizarin Red S which detects calcium deposition from mature osteocytes. Images were captured using a Zeiss Axio Vert200 fluorescence microscope (München, Germany) equipped with a Zeiss Axiocam MRc5 digital camera (München, Germany; Fig. 3).

### Evaluation of GFP expression after differentiation

Adipogenic-, osteogenic- and non-induced cultures were fixed on days 7, 14 and 21 after induction. During the assessment of transduced adipogenic and osteogenic cultures, 4′,6-diamidino-2-phenylindole (DAPI; 0.02 µg/ml) staining was performed. Images were captured using a Zeiss Axio Vert200 microscope equipped with a Zeiss Axiocam MRc5 digital camera, utilizing blue (Excitation G 365; Emission BP 445/50) and green (Excitation BP 450-490; Emission LP 515) filter sets to detect DAPI and GFP, respectively. These photographs were then superimposed on one another using Photoshop Light Room software (photoshop.com).

### Statistical analysis

Non-transduced (control) cells, transduced cells not expressing GFP and transduced cells expressing GFP were assessed with regard to immunophenotype. The means for each replicate were compared using One Way Analysis of Variance across all three groups. The Student’s *T* test was used to compare the means of the transduced cultures expressing GFP and those not expressing GFP with the control non-transduced cells, and a *P* value of <0.05 was considered to be significant.

## Results

Human ASCs were isolated, characterized and expanded to be used for further experiments. All the ASC cultures adhered to the Dominici and co-workers proposed criteria with regard to adherence to plastic, >95 % cell surface expression of CD73, CD90 and CD105, <5 % expression of CD34 and CD45 and differentiation into adipose and osteogenic lineages (Dominici et al. 2006).

A titration study was performed to determine the optimal titer required to transduce the maximum number of ASCs. The vector stock solution contained 22,954 virion particles per microlitre. Low levels of GFP expression were observed across all passages using low volumes (5–100 μl) of viral stock solution. The transduction efficiency improved to more than 50 % GFP expression when the ASCs were exposed to 150 μl (~340,000 virion particles) of the vector stock solution and an optimal transduction efficiency was achieved using 250–300 µl of the vector stock solution. Little difference was observed across 10 post transduction passages with regard to the percentage of GFP expressing cells within the population, when using between 250 and 300 µl of viral stock solution (Table 1). Data at all passages, with the exception of T0 and T3 fitted a Gaussian distribution and the coefficient of determination as determined using the two-tailed Pearson correlation was significant (*R*^2^ = 0.92, *P* < 0.0001). The interpolated values were as follows: 100 % transduction = 363.18 µl; 90 % transduction = 326.86 µl; 80 % transduction = 290.54 µl; 70 % transduction = 254.23 µl (Supplementary Figure 1).

**Fig. 1:**
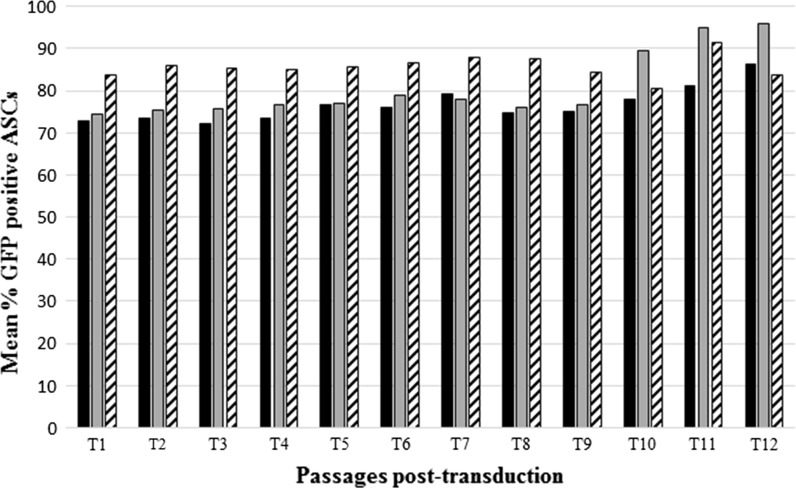
Percentage of ASCs expressing GFP. ASCs were transduced with a GFP-expressing lentiviral vector, and GFP expression was determined by flow cytometry. Data from three biological replicates over 12 consecutive passages post transduction are presented. Values represent mean % GFP positive ASCs

Transduction of three biological replicates (i.e. cells from different donors) was performed using 250 μl lentiviral stock solution with a titer of 22,594 TU/μl and an MOI of ~118. The percentage of ASCs expressing GFP was monitored across 12 consecutive passages (Fig. 1). A stable percentage of GFP expressing cells was observed [mean: 81 %; standard deviation (STD DEV): 4.04] within the transduced cultures over 12 post-transduction passages.

**Fig. 2:**
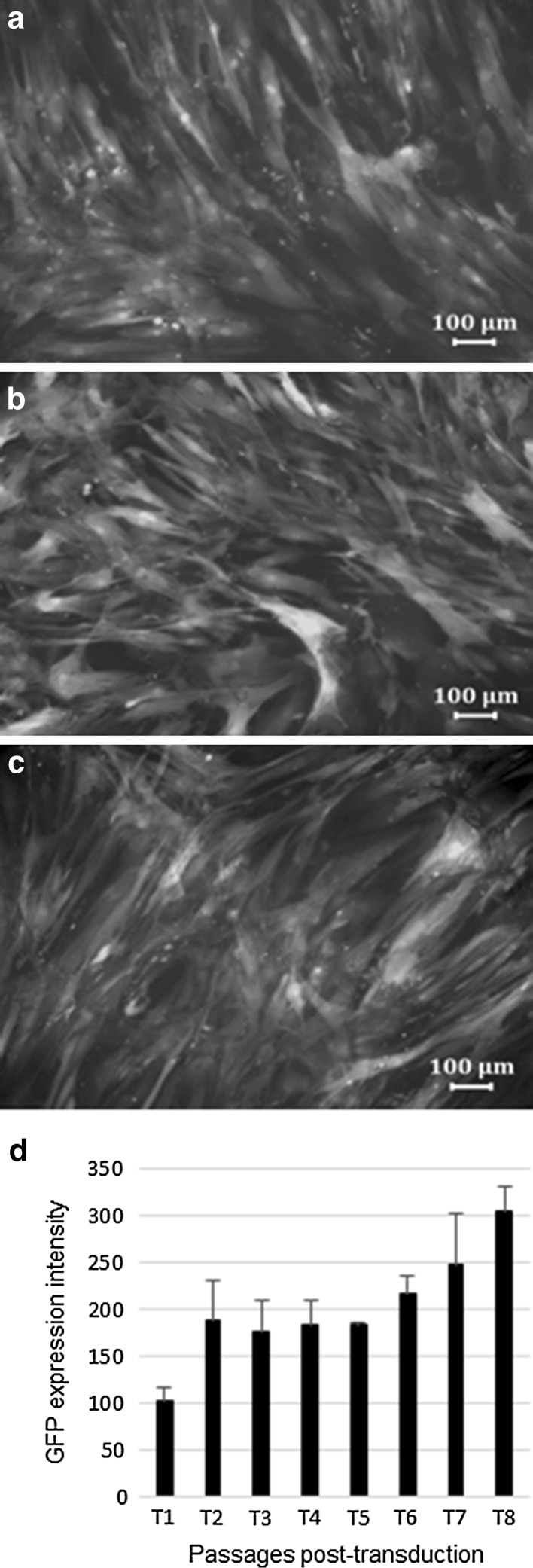
GFP expression intensity across post-transduction passages. **a** ASC culture at post-transduction passage 2 (T2); **b** ASC culture at T8; and **c** ASC culture T14. **a**–**c** are from the same biological replicate (donor). **d** Stable GFP expression intensity (X-GMean) was observed across eight post-transduction passages (T) using flow cytometry. A slight increase in GFP expression intensity can be observed with increasing post-transduction passages. Values are mean ± STD DEV from three pooled donor cultures

**Fig. 3:**
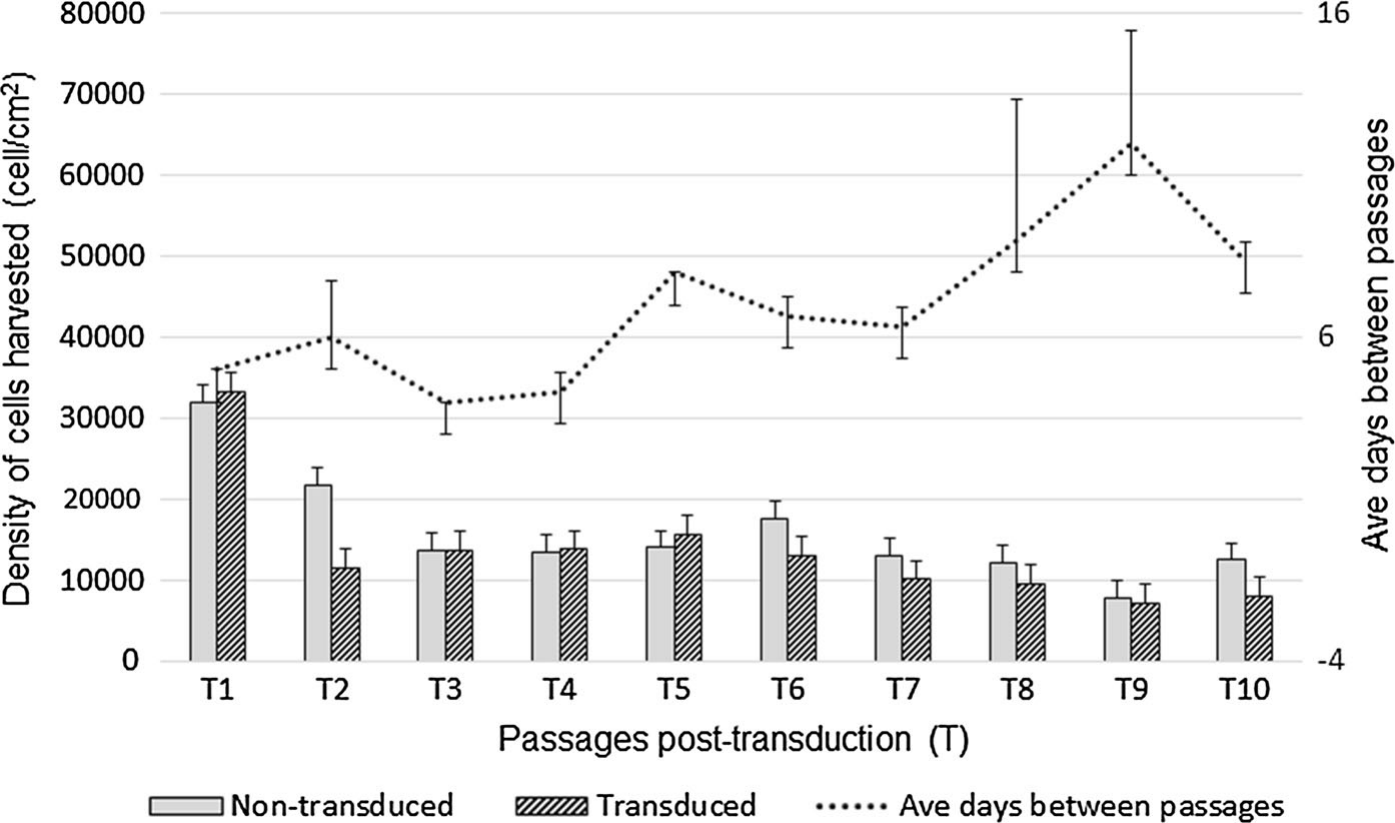
Proliferation of non-transduced and transduced ASCs across passages T1–T10. For every biological replicate a non-transduced as well as a transduced culture was seeded and harvested on the same day at 70–80 % confluency. The *left axis* represents the density of cells harvested and is shown as cells/cm^2^. The *right axis* indicates the mean number of days the cultures were left to expand from seeding to harvesting at 70–80 % confluency. Values are mean ± STD DEV

Fluorescence (expression) intensity was assessed using flow cytometry and microscopic imaging at post-transduction passages (T). Sustained GFP expression intensity was observed across 14 post-transduction passages. Visual images at T2, T8 and T14 showed little difference in fluorescence intensity (Fig. 2a–c), while flow cytometry results quantitatively measured a slight increase in fluorescence intensity with increasing passages (Fig. 2d; One outlier at T5 was removed from the analysis). Little difference was observed when comparing the mean proliferation of non-transduced and transduced ASCs across 10 passages T1–T10 (Fig. 3). Every biological replicate had a non-transduced as well as a transduced culture that was seeded and harvested on the same day at 70–80 % confluency. The number of cells harvested at every passage is expressed as cell density (cells/cm^2^). The number of days between passages increased with increasing passages, suggesting a decrease in proliferation capacity with increasing passage number.

The cells were analyzed on a Gallios flow cytometer using a multiparameter approach. Cells were simultaneously stained with CD105 PE (FL 2), CD90 PC5 (FL 4), CD34 PC7 (FL5), CD73 BV510 (FL9) and CD45 KO (FL10). GFP (FL 1) expression was also assessed. Colour compensation was set according to single colour staining tubes and appropriate Fluorescence-Minus-One (FMO) control tubes were used to ensure optimal colour compensation and region of interest settings. We observed that non-transduced and transduced cultures displayed similar phenotype profiles, when the co-expression of the markers was considered. However, sub-dividing the transduced culture into GFP positive cells (successfully transduced) and GFP negative cells (not successfully transduced), showed a trend towards a decrease, although not significant, in the immunophenotypic co-expression within the GFP positive transduced cells (Table 2).

**Table 2:** GFP expression and CD34 −, CD45 −, CD73 +, CD90 +, CD105 + immunophenotype in ASCs transduced with a GFP

Biological replicate	GFP expression across 12 passages^a^		CD34 −, CD45 −, CD73 +, CD90 +, CD105 + immunophenotype across 4 passages^b^								*P* value comparison across all groups^c^ (GFP positive, negative and non-transduced)
			Non-transduced culture (control)		Transduced culture GFP negative			Transduced culture GFP positive			
	GFP+	GFP-	Mean	STD DEV	Mean	STD DEV	*P* value compared to control	Mean	STD DEV	*P* value compared to control	
1	77	23	93.70	4.88	91.31	5.50	0.28	86.51	6.15	0.082	0.34
2	81	19	76.00	19.88	66.08	27.75	0.30	55.02	18.41	0.11	0.55
3	86	14	67.75	24.85	64.93	31.34	0.45	40.1	35.84	0.15	0.66
Mean	81	19	79.15	13.261	74.10	14.91	0.35	60.54	23.69	0.18	0.5

^a^Mean % GFP positive and negative cells across 12 passages

^b^Values represent mean ± STD DEV of ASCs expressing the CD34 −, CD45 −, CD73 +, CD90 +, CD105 + immunophenotype across four passages: T2-T5 for replicate 1; T8-T11 for replicate 2; T16-T19 for replicate 3

^c^ *P* values were determined by a three way comparison between non-transduced, transduced GFP negative and transduced GFP positive cultures

**Fig. 4:**
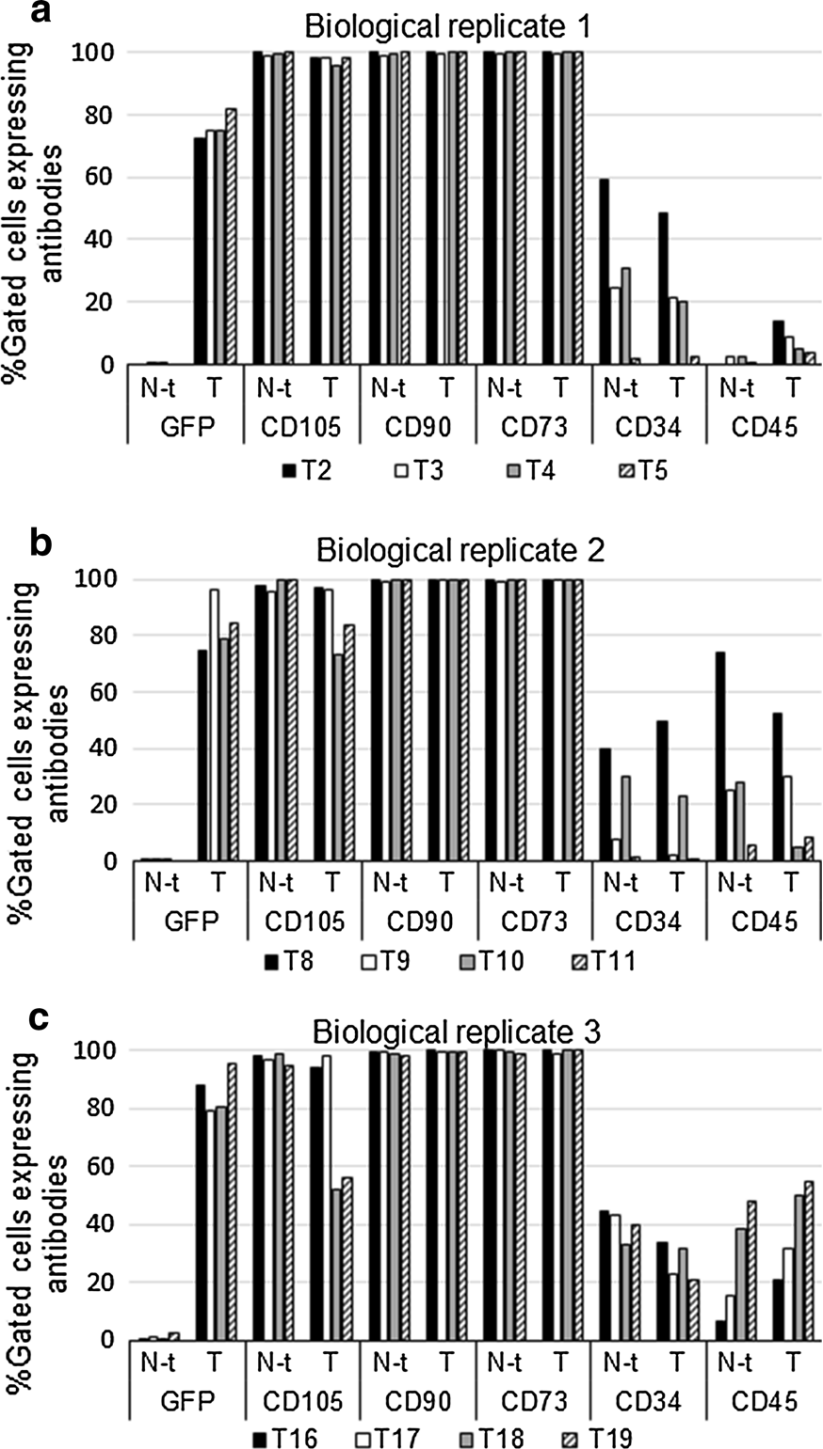
Expression profile of individual markers in transduced ASCs from three biological replicates (donors). Expression of cell surface markers CD 105 PE (FL 2), CD 90 PC5 (FL 4), CD 34 PC7 (FL5), CD73 BV510 (FL9) and CD45 KO (FL10) was detected by flow cytometry. GFP (FL 1) is indicated as a percentage of the gated ASC population. The individual marker expression profiles are shown individually as **a**–**c** over four consecutive post-transduction passages namely T2–T5, T8–T11 and T16–T19 for three biological replicates, respectively. Every biological replicate had one GFP lentiviral vector transduced culture (represented by T on *x*-axis) and one non-transduced culture (represented by *N* − *t* on *x*-axis) that were processed similarly. Cells were seeded at a density of 5000 cells/cm^2^ and were harvested on the same day at 70–80 % sub-confluency

In order to establish if specific markers are responsible for the observed change in the co-expression profile of the GFP positive cells, the expression of individual markers was also investigated (Fig. 4). The individual marker expression profiles of three biological replicates is shown individually as (a), (b) and (c) over four consecutive post-transduction passages, namely T2–T5, T8–T11 and T16–T19 for biological replicates 1, 2, and 3, respectively (Fig. 4). Thus the biological replicates were assessed at the same post-transduction passages. Similarities were observed with regard to the expression profiles of the individual markers between the non-transduced and transduced cultures at respective post transduction passages. We did however observe a decrease in CD105 in biological replicates 2 and, in particular, 3 with increasing passages, as well as varying levels of expression of CD34 and CD45 (no consistent pattern) in all non-transduced and transduced cultures. The trend in replicates 1 and 2 was a decrease in non-ASC markers, while the outstanding trend in replicate 3 was an increase in CD45. The reasons or these differences are not known. In view of the increase in CD45 in replicate 3, we would choose not to use these cells for tracking purposes.

**Fig. 5:**
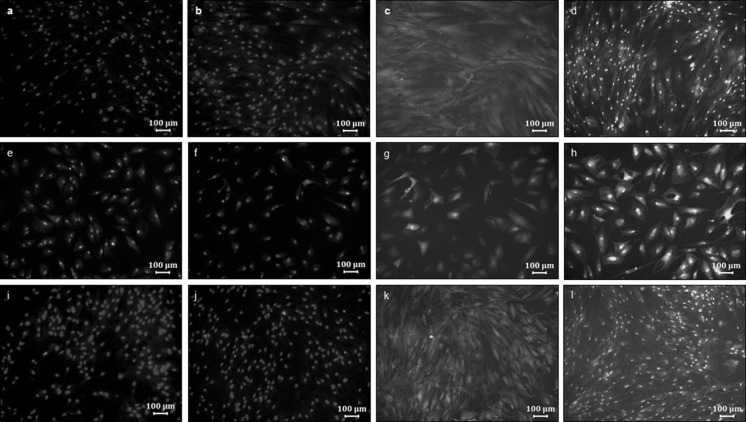
Fluorescence microscopy analysis of adipogenic and osteogenic differentiation in non-transduced and transduced cultures. **a** Non-transduced, non-induced ASCs, stained with DAPI. **b** Transduced, non-induced ASCs, stained with DAPI. **c** Transduced, non-induced ASCs, with a GFP positive cytoplasm. **d** Transduced, non-induced ASCs, with overlay of the DAPI stained and GFP expression images (different vision field than **b** and **c**). **e** Non-transduced, adipogenic induced ASCs, stained with DAPI. **f** Transduced, adipogenic induced ASCs, stained with DAPI. **g** Transduced, adipogenic induced, GFP positive cytoplasm. **h** Transduced, adipogenic induced ASCs, with overlay of the DAPI stained and GFP positive expression images (different vision field than **f** and **g**). **i** Non-transduced, osteogenic induced ASCs, stained with DAPI. **j** Transduced, osteogenic induced ASCs, stained with DAPI. **k** Transduced, osteogenic induced ASCs, GFP positive cytoplasm. **l** Transduced, osteogenic induced ASCs, with overlay image of DAPI stained and GFP positive expression images (different vision field than **j** and **k**). These representative images are of the day 21 post-induction time point

**Fig. 6:**
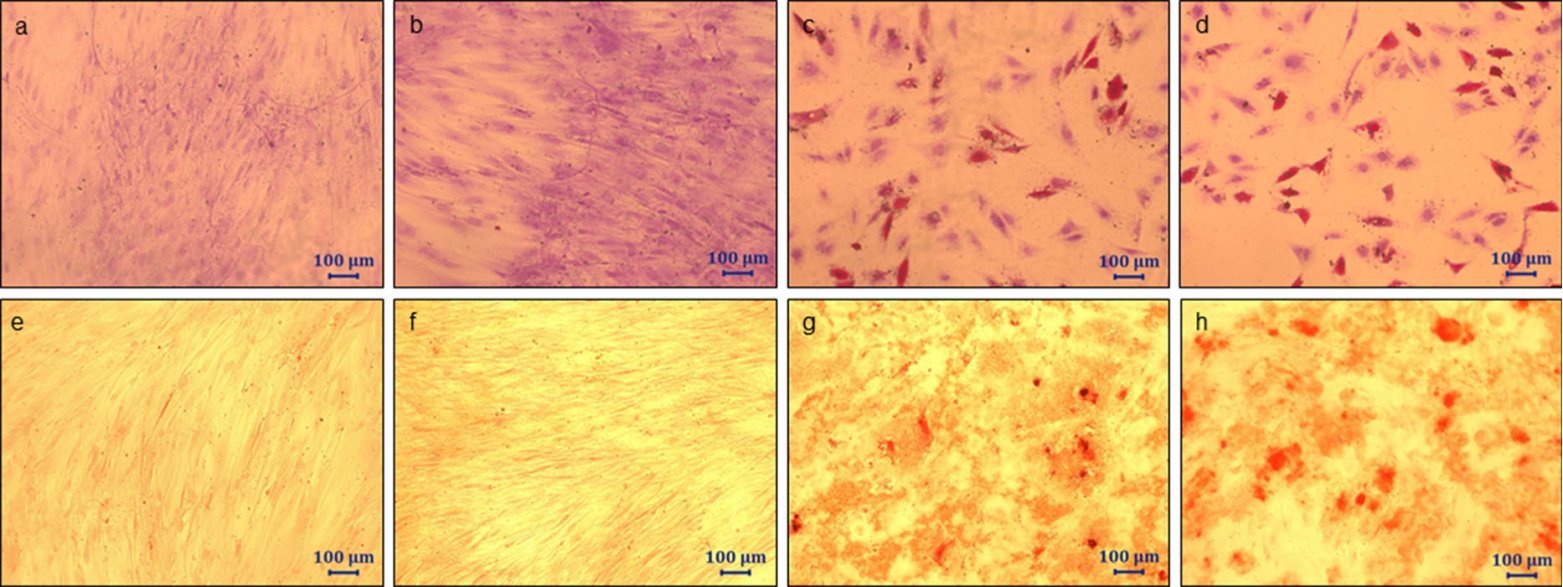
Qualitative microscopic assessment of adipogenic and osteogenic induced cultures accompanied by their respective non-induced controls. Oil Red O stained lipid droplets confirm adipogenic differentiation and Alizarin Red S positive staining confirms calcium deposition following osteogenic differentiation in both non-transduced and transduced cultures. **a** Non-transduced, non-induced ASCs, stained with Oil Red O and counter stained with 1 % Toluidine Blue. **b** Transduced, non-induced ASCs, stained with Oil Red O and counter stained with 1 % Toluidine Blue. **c** Non-transduced, adipogenic induced ASCs, stained with Oil Red O and counter stained with 1 % Toluidine Blue. **d** Transduced, adipogenic induced ASCs, stained with Oil Red O and counter stained with 1 % Toluidine Blue. **e** Non-transduced, non-induced ASCs, stained with Alizarin Red S. **f** Transduced, non-induced ASCs, stained with Alizarin Red S. **g** Non-transduced, osteogenic induced ASCs, stained with Alizarin Red S. **h** Transduced, osteogenic induced ASCs, stained with Alizarin Red S. These representative images are of the day 21 post-induction time point

Non-transduced and transduced cells of the three individual cultures were induced to differentiate into adipogenic and osteogenic lineages in vitro. The lineages were confirmed using Oil Red O to indicate the presence of intracellular lipid droplets during adipogenic differentiation and Alizarin Red S to detect calcium deposits following osteogenic differentiation (Figs. 5, 6).

From the DAPI stained images it was clear that the cells were “over populated”, especially with regard to the transduced, non-induced controls and the osteogenic induced cultures. The GFP images for these respective cultures showed overlaying bright expression making it difficult to locate individual cells. Individual GFP positive cells could be observed in the transduced, adipogenic induced cultures (Fig. 5).

Both non-transduced and transduced cultures were able to differentiate into adipocytes as visualized by Oil Red O stained intracellular lipid droplets and also into osteogenic tissue as evidenced by the presence of Alizarin Red S stained calcium deposits (Fig. 6). It was noticed that the density of adherent cells decreased considerably during adipogenic induction; this was observed in both non-transduced and transduced cultures.

## Discussion

This study shows successful transduction of human ASCs with a lentiviral vector encoding GFP for potential use as a tracking system in future in vivo studies. Our results also show sustained expression of GFP during extensive expansion (>10 passages), as well as adipogenic and osteogenic differentiation in vitro.

The titration study showed stable expression of GFP following transduction with 250 μl of virus stock solution, which translates to a titer of 22,594 TU/µl and an MOI of ~118. The process of preparing GFP positive lentiviral stock is time consuming and expensive and it is therefore important to consider using smaller volumes of the virus stock solution for transduction. However, according to our findings, a virion to cell ratio of less than 25:1 would not result in optimal transfection and is therefore likely to result in suboptimal assessment of the homing abilities/transplant efficiency of the transduced cells, as a large portion of the transplanted cells would not be able to be tracked. This problem could be overcome by selection for GFP-positive cells using fluorescent-activated cell sorting (FACS) prior to in vivo transplantation.

Although more than 70 % of the cells expressed GFP at all time-points, the expression varied among the three biological replicates (donors), one being consistently more than 80 % and the other showing increased expression from passage eight onwards, making this marker a suitable candidate for future expansion experiments. The difference in GFP expression observed between different biological replicates could be due to inter-patient variability.

All the transduced and non-transduced cultures differentiated successfully into adipogenic and osteogenic lineages. Overlaying DAPI stained and GFP images served as a quality control for GFP expression. All cells within the adipogenic induced cultures demonstrated GFP expression. Interestingly, the adipogenic induced cells did not expand to confluency compared to the osteogenic induced cells, and this seems to be a consistent finding. Possible reasons include: (a) initial cell death during induction due to toxicity of the induction medium; (b) the nature of adipocytes which are “contact inhibited” and require a specific surface area to expand; or (c) only a specific sub-population within the heterogeneous population has the ability to differentiate into the adipogenic lineage, although the findings are consistent for both transduced and non-transduced cultures. Also, from the overlaid images, one can observe matrix formation (blurry effect in fluorescent images) of the osteogenic induced cultures which were also GFP positive.

An important finding is that we did not observe a decrease in GFP expression during the differentiation process. This is important when considering using this marker to investigate homing and site-specific differentiation of ASCs in pre-clinical applications in animal models. Similar findings of GFP expression stability in umbilical cord derived MSCs were reported by Tao et al. (2014) confirming that lentiviral vector mediated GFP expression provides a stable, efficient labeling model in comparison to other tracking models including 5-bromo-2-deoxyuridine (BrdU) and DAPI. Models utilizing BrdU and DAPI present with a number of disadvantages such as quenching during cell division as well as quenching over long time frames. In addition, GFP lentiviral vector labeling does not require expensive reagents and detection equipment as does Y chromosomal labeling and magnetic tagging (Tao et al. 2014).

We were able to obtain more than 80 % lentiviral transduction efficiency of ASCs. No significant changes in proliferation capacity or in cell surface marker expression were observed between transduced and non-transduced cultures, although in one biological replicate (no. 3) there was an increase in CD45 with increasing passage number. The reasons for this increase are not know, but we would exclude these cells from further use for tracking purposes. Transduced cells like non-transduced cells demonstrated similar morphology and differentiation.

The percentage GFP positive cells seem to decrease slightly during passages 1 and 5, suggesting a transition phase, followed by a steady increase thereafter. The level of GFP expression in individual cells did not decrease with time, demonstrating persistent intracellular GFP protein production. These optimized GFP positive lentiviral vector transduction procedures and our results obtained therefrom demonstrate that the standardized criteria used for defining human ASCs were not compromised. Future research will be directed at ASC tracking to study homing, migration, engraftment and in situ differentiation, all of which are relevant to organ regeneration and restoration. To use this ASC lentiviral transduction technique for gene delivery applications, further transduction optimization will be needed to obtain a minimal number of copies per transduced cell.

## Electronic supplementary material

Below is the link to the electronic supplementary material.

Supplementary Figure 1 Gaussian distribution curve of GFP positive transduced cells. Adherent ASCs (48 000 cells) were transduced with different dilutions of lentiviral vector stock solutions and GFP expression was measured over 10 post-transduction passages using flow cytometry. The following amounts of lentivirus vector stock solution - 0-, 5-, 10-, 25-, 50-, 100-, 150-, 200-, 250- and 300 µl – were utilized to determine the optimal titer for ASC transduction. Data at all post transduction passages (T), with the exception of T0 and T3 fits with the Gaussian distribution curve. The interpolated value for 100 % transduction was found to be 363.18 µl lentiviral vector stock solution. (TIFF 130 kb)

Supplementary Figure 2 Overlaying plots comparing the unstained control, non-transduced and transduced GFP positive cells for individual markers. The plots are from one biological replicate at a specific post-transduction passage. Each plot displays the expression of an individual antibody marker or GFP expression in the cell cytoplasm. (A) CD73 BV510; (B) CD90 PC5; (C) CD105 PE; (D) CD34 PC; (E) CD45 KO; and (F) GFP. (TIFF 388 kb)

## Abbreviations

ASC: Adipose derived stromal/stem cell
GFP: Green fluorescent protein
HIV-1: Human immunodeficiency virus type-1
SVF: Stromal vascular fraction
pen/strep: Penicillin and streptomycin
FBS: Fetal bovine serum
α-MEM: Alpha-Modified Eagle Medium GlutaMAX™
DMEM: Dulbecco’s Modified Eagle Medium
PBS: Phosphate buffer saline
HBS: Hank’s Balanced Salt Solution without Ca_2_^+^Mg_2_^+^
MOI: Multiplicity of infection
TU/µl: Transducing units per microliter
ddH_2_O: Double distilled water
STD DEV: Standard deviation
