# Supplementary figures and images for: Human adipose derived mesenchymal stromal cells transduced with GFP lentiviral vectors: assessment of immunophenotype and differentiation capacity in vitro
Source: Cytotechnology. 2016 Jan 27;68(5):2049–60. doi: 10.1007/s10616-016-9945-6 (PMC5023578; doi:10.1007/s10616-016-9945-6)

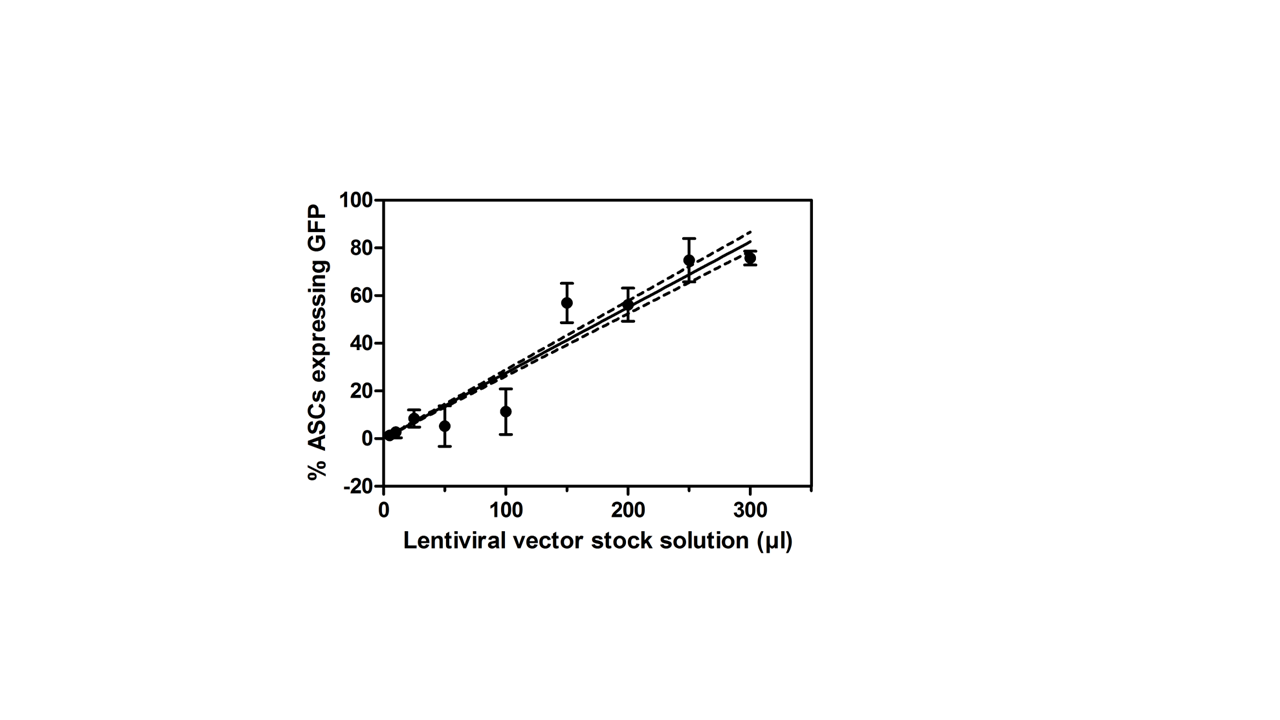

Supplement: Supplementary file 1 — Supplementary Figure 1 Gaussian distribution curve of GFP positive transduced cells. Adherent ASCs (48 000 cells) were transduced with different dilutions of lentiviral vector stock solutions and GFP expression was measured over 10 post-transduction passages using flow cytometry. The following amounts of lentivirus vector stock solution - 0-, 5-, 10-, 25-, 50-, 100-, 150-, 200-, 250- and 300 µl – were utilized to determine the optimal titer for ASC transduction. Data at all post transduction passages (T), with the exception of T0 and T3 fits with the Gaussian distribution curve. The interpolated value for 100 % transduction was found to be 363.18 µl lentiviral vector stock solution. (TIFF 130 kb) [file 10616_2016_9945_MOESM1_ESM.tif]

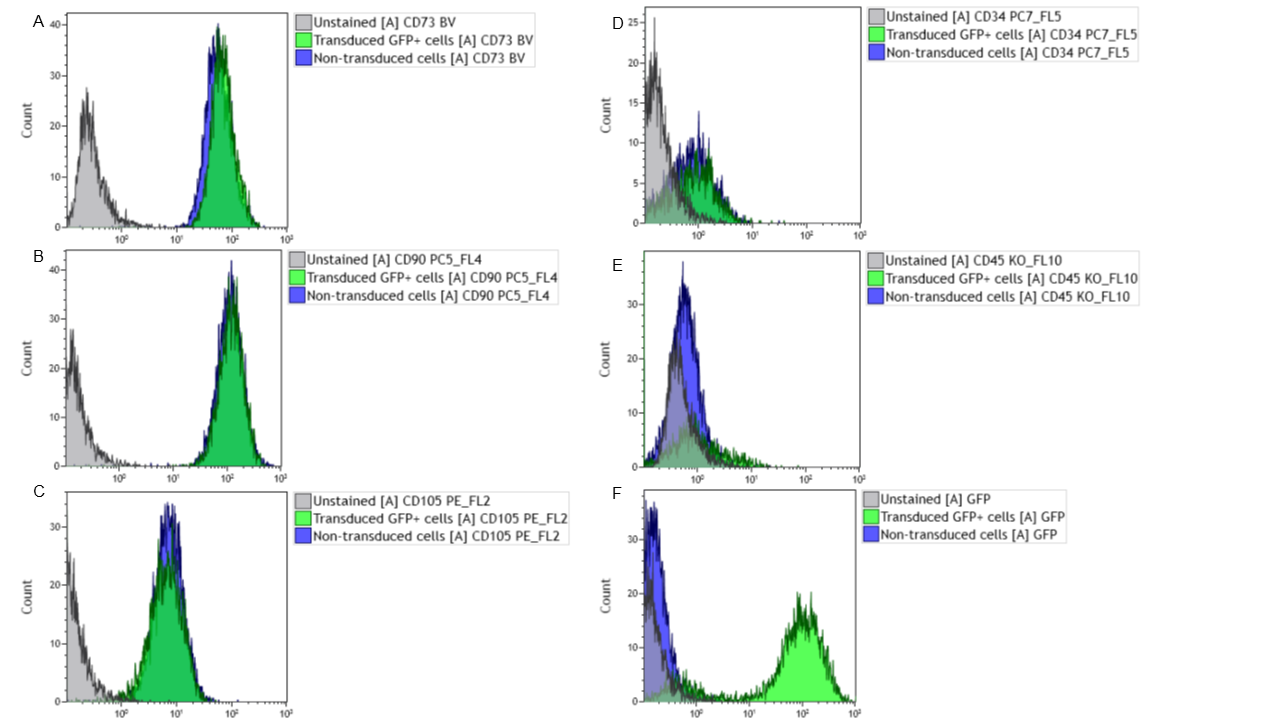

Supplement: Supplementary file 2 — Supplementary Figure 2 Overlaying plots comparing the unstained control, non-transduced and transduced GFP positive cells for individual markers. The plots are from one biological replicate at a specific post-transduction passage. Each plot displays the expression of an individual antibody marker or GFP expression in the cell cytoplasm. (A) CD73 BV510; (B) CD90 PC5; (C) CD105 PE; (D) CD34 PC; (E) CD45 KO; and (F) GFP. (TIFF 388 kb) [file 10616_2016_9945_MOESM2_ESM.tif]
